# Automated seismic fault mapping using convolutional neural networks for the Berenice Field, Western Desert, Egypt

**DOI:** 10.1038/s41598-026-66912-4

**Published:** 2026-08-20

**Authors:** Mohammed Amer, Walid M. Mabrouk, Amr M. Eid, Ahmed Metwally

**Affiliations:** Geophysics Department, Faculty of Science, Cairo University, Giza, 12613 Egypt

**Keywords:** User-trained fault prediction, CNN, Machine learning (ML), Seismic interpretation, Berenice Field, Mathematics and computing, Solid Earth sciences

## Abstract

Accurate fault interpretation remains a cornerstone of hydrocarbon exploration, directly influencing structural modeling, reservoir compartmentalization, and well placement decisions. However, conventional manual fault picking is time-intensive and prone to interpreter bias, especially in structurally complex and noisy seismic datasets such as those from Egypt’s Western Desert. This study demonstrates the implementation and performance of Schlumberger’s user-trained Convolutional Neural Network (CNN) based Fault Prediction Module within the Petrel E&P platform for automated fault detection in the Berenice Field, Faghur basin. The applied workflow integrates machine learning–based denoising, sparse interpreter-guided fault labeling, 3D probability cube generation, and fault extraction using thresholded planarity and azimuth attributes. Only three manually labeled seismic lines (< 1% of total volume) were sufficient for model training. The CNN model achieved a Dice coefficient exceeding 0.85 and successfully delineated 65% of the manually mapped faults, while also detecting additional previously unmapped subtle discontinuities. The ML-assisted workflow reduced interpretation time by over 80% compared to traditional methods, maintaining high geological coherence and consistency. These results confirm that machine learning–driven fault detection effectively enhances interpretational accuracy and efficiency in tectonically complex domains. The successful application to the Berenice Field underscores the potential of CNN-based fault prediction as a transformative tool for structural interpretation across Egypt’s Western Desert and similar intracratonic settings worldwide.

## Introduction

Seismic data interpretation remains a foundational pillar of hydrocarbon exploration and production, underpinning accurate reservoir delineation, risk assessment, and optimal well placement strategies. Faults, representing tectonic discontinuities that juxtapose rock strata and influence fluid migration pathways, often appear as faint amplitude anomalies or reflector terminations within 3D seismic volumes. Despite advances in acquisition technology yielding high-fidelity datasets, manual fault picking, traditionally executed via horizon auto trackers and semblance attributes remains labor-intensive, subjective, and error-prone, especially across expansive survey areas exceeding thousands of square kilometers^1,2^.

The integration of machine learning (ML) into seismic workflows heralds a transformative era, shifting paradigms from exhaustive human-led delineation to probabilistic, data-driven predictions. Leveraging convolutional neural networks (CNNs), these systems ingest sparse interpreter-provided fault labels to train patch-based classifiers, extrapolating fault likelihood across entire seismic cubes with sub-millisecond inference speeds. This automation not only curtails interpretation cycles from weeks to mere hours but also mitigates cognitive biases inherent in manual workflows, fostering consistency across multidisciplinary teams^3,4^.

Schlumberger’s Delfi subsurface platform, through its advanced Petrel E&P software suite, operationalizes this capability via the ML Fault Prediction module a cornerstone of its structural interpretation toolkit. Practitioners initiate the process by digitizing minimal fault evidence (e.g., 10–20 sticks per fault) as training exemplars, prompting the CNN to output a fault probability volume. Visualized via dynamic opacity thresholds (e.g., 0.5 probability isosurfaces rendered in spectral colormaps), these predictions facilitate semi-automated extraction of fault point clouds, stick vectors, and triangulated surfaces, fully interoperable with horizon frameworks and geocellular models^5,6^.

Empirical validations, such as those chronicled in SPE-202,930-MS and analogous North Sea basin studies, affirm the module’s efficacy in resolving subtle low-throw faults (< 15 ms two-way time) obscured by overburden multiples or anisotropic velocity artifacts. Quantitative benchmarks reveal Dice coefficients exceeding 0.85 for fault predictions versus ground-truth labels, surpassing conventional coherence-based attributes by 20–30% in F1-scores. This precision enables iterative refinement loops, where interpreters upscale training data to hone model outputs, culminating in robust multi-realization uncertainty frameworks^7,8^.

The technology’s versatility extends to frontier applications, including salt-flank fault imaging, carbonate fracture corridor mapping, and geothermal reservoir screening, where integration with seismic inversion attributes (e.g., AVO-compliant probability fusion) unveils sub-seismic scale features critical for containment analysis. In mature fields, it accelerates history-matched fault transmissibility modeling, informing enhanced oil recovery pilots with reduced dry-hole risks. Schlumberger’s cloud-native scalability within Delfi further democratizes access, enabling collaborative interpretation across global asset teams, with this article specifically evaluating its performance on seismic datasets from Egypt’s prolific Western Desert province—focusing on the Bernice Field as a representative case study of complex fault regimes in clastic reservoirs^9,10^.

This article elucidates the theoretical underpinnings, implementation protocols, and quantifiable impacts of Schlumberger’s automated fault detection paradigm, with a primary focus on its application to Egypt’s seismic datasets from the Western Desert, particularly the complex structural regimes of the Bernice Field. Characterized by intricate fault networks, subtle low-throw displacements, and clastic reservoir heterogeneities influenced by multiphase tectonics, these datasets pose significant challenges for conventional interpretation methods. By synthesizing peer-reviewed case studies like SPE-202,930-MS with targeted ML Fault Prediction workflows on Bernice Field volumes, the analysis demonstrates enhanced resolution of sub-seismic faults critical for reservoir compartmentalization and fluid flow modeling^11,12^.

The goal centers on validating the efficacy of CNN-driven auto-fault detection in resolving Egypt’s geologically demanding structures, where overburden multiples and anisotropic effects often obscure key discontinuities. Through iterative training on sparse local labels, probability volume generation, and semi-automated stick extraction within Petrel, the study quantifies improvements in Dice coefficients (> 0.85) and interpretation speed, directly addressing regional complexities like fault juxtaposition seals in mature fields. These insights equip geoscientists with practical strategies to deploy Schlumberger’s Delfi tools for high-fidelity structural models, reducing exploration risks in analogous tectonically active basins^3^.

## Geological setting

**Fig. 1:**
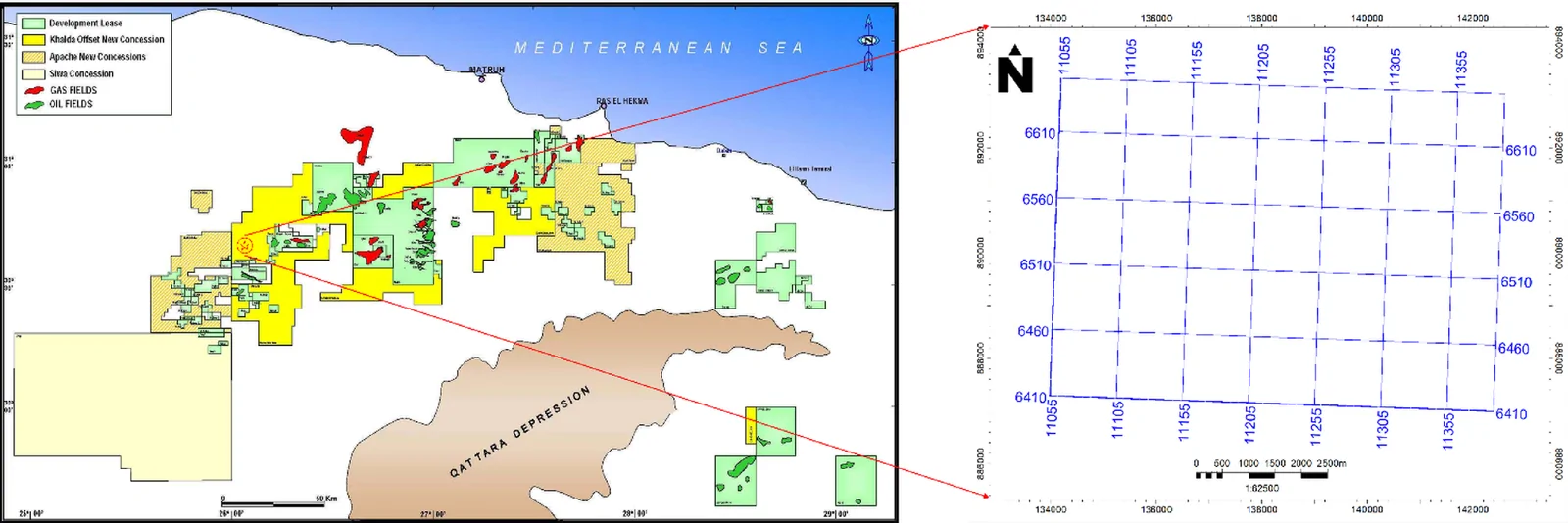
Map of the study area showing the seismic lines and the location of the Berenice Field^16^.

The Berenice Oil Field is located in the northeastern sector of the Faghur Basin within Egypt’s Western Desert, an intracratonic sedimentary province that records a prolonged and complex tectono-sedimentary evolution throughout the Mesozoic and Cenozoic (Fig. 1). The basin architecture was established during early Mesozoic rifting associated with the break-up of Gondwana, which initiated significant extensional tectonics and the formation of normal faults that dominate the field’s structural framework. Subsequent tectonic events, including Late Cretaceous to Paleogene inversion related to the Syrian Arc deformation, further modified this architecture, producing varied fault orientations and reactivation histories that influence trap styles and reservoir compartmentalization^13,14^.

Structurally, the field is characterized by an interplay of normal fault systems trending primarily ENE–WSW, NW–SE, and subordinate NE–SW directions. The dominant ENE–WSW faults displace Paleozoic and Mesozoic sequences, creating a series of horst and graben blocks. These step-faulted configurations are observed in seismic sections and are responsible for lateral segmentation of reservoir packages, forming structural highs and lows that serve as hydrocarbon traps and influence fluid distribution. The NW–SE and NE–SW sets complement this framework, further defining reservoir boundaries and compartmentalization^15–17^.

The prevalence of normal faulting reflects regional extensional stress regimes that controlled deposition and structural relief during the Jurassic and Early Cretaceous. Fault throws vary laterally, and their orientations often define the limits of reservoir compartments, particularly within the Alam El Bueib Formation and its AEB‑3E reservoir unit. The ENE–WSW and NW–SE sets form the primary structural grain, with displacement creating opportunities for both fault-block traps and juxtaposition against sealing lithologies. While dip-slip motion dominates, subtle oblique and locally reactivated segments indicate minor variations in kinematics during subsequent deformation phases^13,18,19^.

This intricate geologic framework, marked by polyphase faulting and heterogeneous reservoirs, positions the Berenice Field as an ideal testbed for automated fault detection, linking tectonic history with seismic interpretation challenges. In addition to extensional structures, Late Cretaceous inversion and minor transpressional features locally modified fault kinematics, influencing trap integrity and seal effectiveness. In this study, machine learning based fault prediction is applied to the Berenice Field, providing a methodology that can be extended to other fields across the Western Desert^14,19–21^.

Applying automated fault detection in Berenice Field holds paramount importance given its polyphase fault networks and subtle discontinuities that confound manual workflows. Schlumberger’s ML Fault Prediction module excels here by resolving low-throw faults obscured by multiples, enhancing reservoir compartmentalization models, and quantifying seal uncertainties for optimized well placement and EOR strategies^22,23^. This approach accelerates interpretation in such tectonically complex settings, reducing risks in Egypt’s Western Desert plays^13^.

## Material and methodology

This study utilizes the Schlumberger workflow for automated fault detection, progressing from data management through denoising, model training, and final fault extraction within the Petrel platform. The primary input consists of full-stack time-domain onshore seismic volumes, approximately 9 × 8 km in size, from the Berenice Field. To maintain structural fidelity amid the Western Desert’s complex polyphase tectonics. Although both pre-trained and user-trained fault prediction models are available within Schlumberger’s framework, this investigation focuses exclusively on user-trained models, which demonstrate superior performance for locally-specific fault characteristics. These models are guided by interpreter-provided fault labels and horizon seeds that direct the CNN learning process toward geologically relevant features^3,10^.

**Fig. 2:**
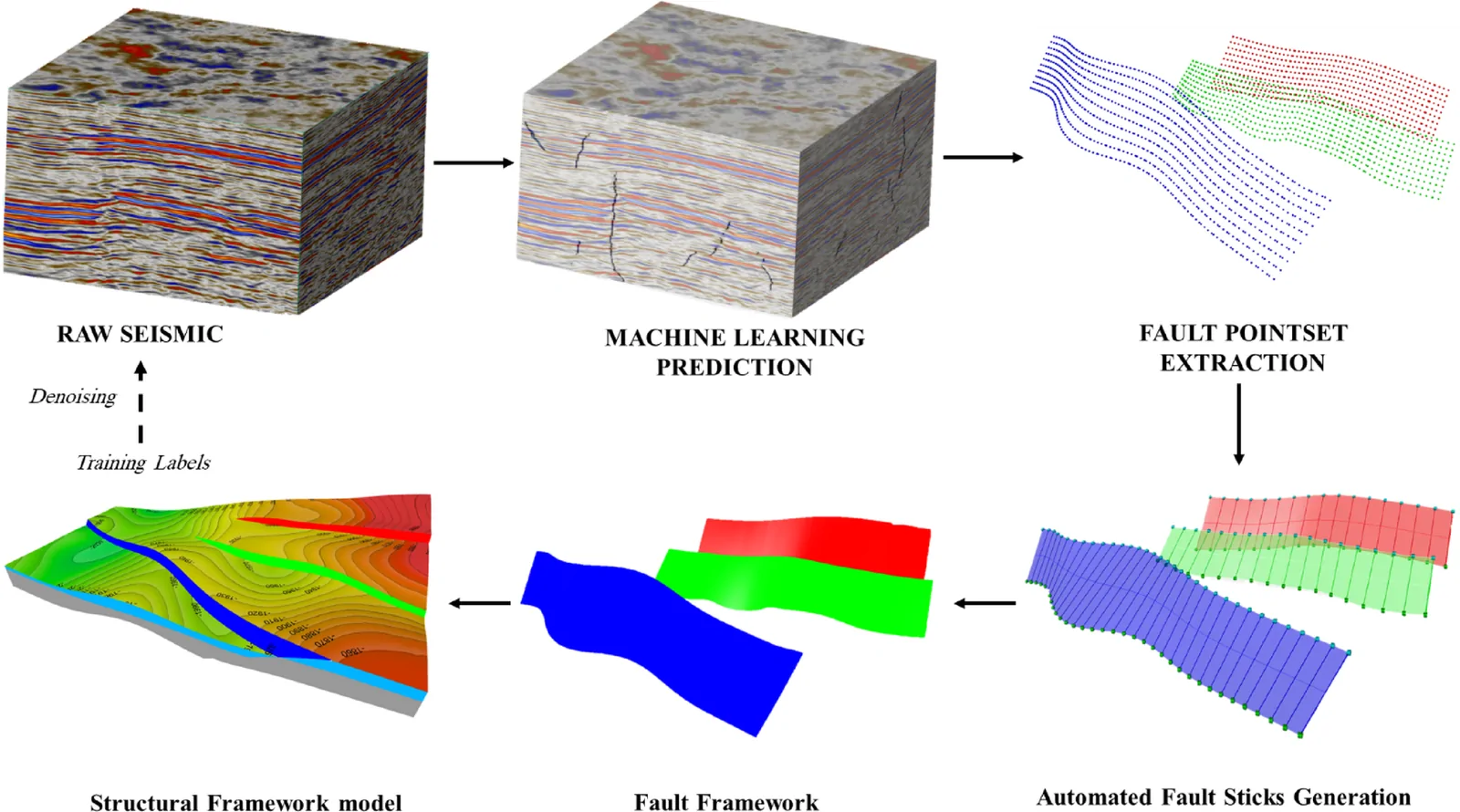
Machine learning-assisted fault interpretation workflow.

The machine learning assisted fault interpretation workflow (Fig. 2) follows a sequence of interdependent stages designed to ensure geological reliability. The process begins with training label definition, followed by ML-based seismic denoising to enhance signal clarity and remove acquisition artifacts (e.g., multiples) that could mask subtle discontinuities. This preprocessing is critical as it prepares the seismic volume for the CNN, ensuring that the subsequently generated fault probability cube focuses on genuine structural features rather than background noise^3,11^.

**Fig. 3:**
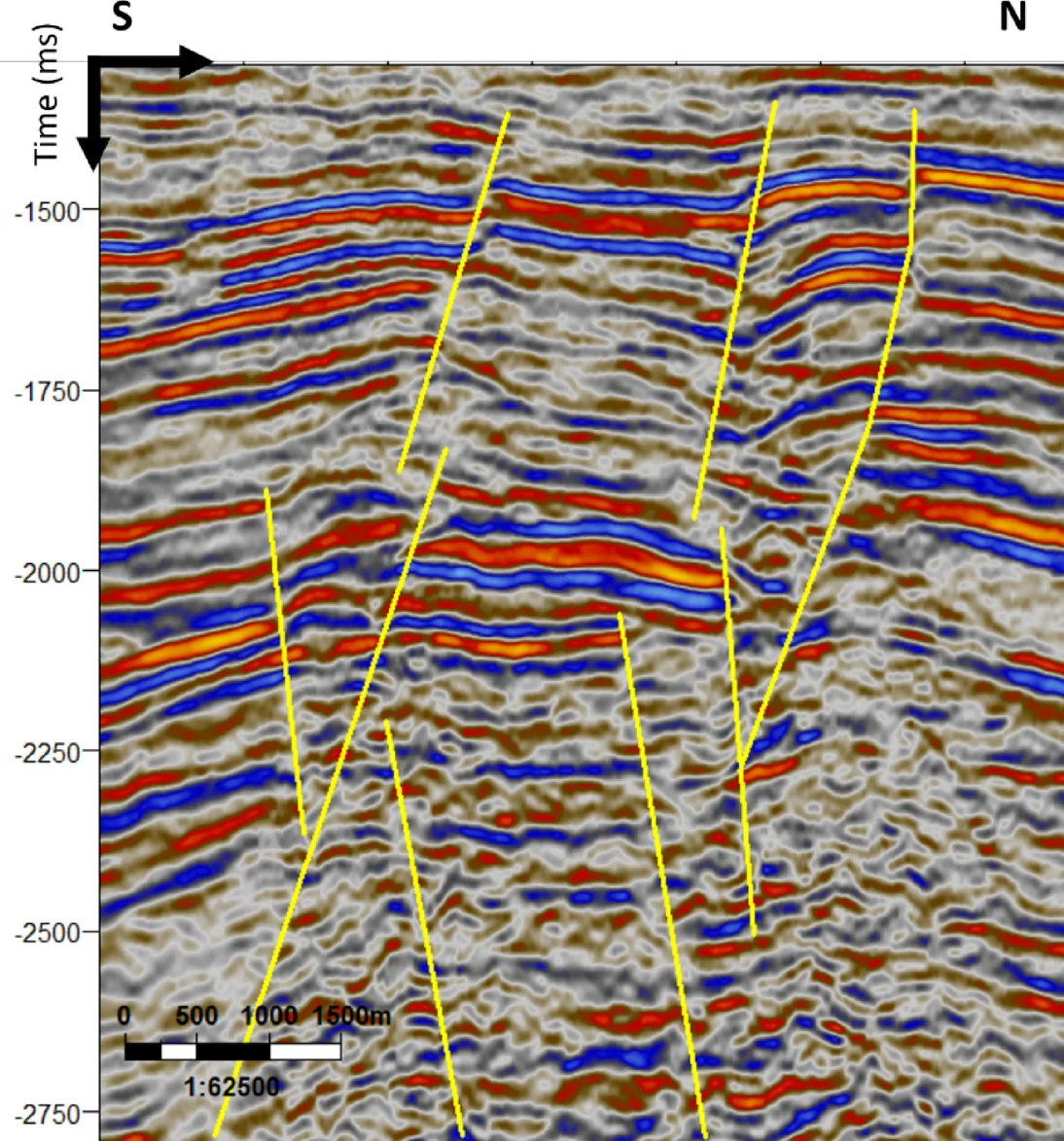
Manually picked seismic faults provided as training labels to guide machine-learning fault prediction.

The first and most fundamental phase is the definition of **training labels** (Fig. 3). Fault labels consist of sparsely interpreted, unassigned fault sticks provided by the interpreter and used by the ML system to characterize the appearance of faults within the seismic volume from the interpreter’s viewpoint. The ML system is trained on these input labels and then generates fault predictions for every pixel throughout the entire seismic volume. In this study, fault training labels were picked on three seismic lines, accounting for less than 1% of the total available lines. The selection of training lines (the seismic sections on which labels were created) was based on data quality, signal-to-noise ratio, frequency content, and the degree of structural variability observed in the seismic dataset. To train the ML system effectively, the training labels must capture the structural variability present in the volume so that the system can reliably identify the targeted structures. Consequently, greater structural complexity requires a larger number of training labels. Manual fault picking is carried out on a strategically selected subset of 2D lines and crosslines that collectively represent the range of structural styles in the study area, including ENE–WSW faults, NW–SE lineaments, and subtle low-throw discontinuities less than 15 ms two-way travel time. Although these sections cover only part of the full seismic volume, they are selected so that they capture all relevant variations in fault morphology and therefore provide the convolutional neural network with explicit examples of true fault signatures. Consistency during this stage is essential. If a fault is mapped on one line but omitted along its clear continuation on adjacent lines, the model receives contradictory instructions and may interpret the same feature both as fault and non-fault. To avoid such systematic bias, complete interpretations are performed along representative lines, ensuring that the network correctly learns the geometry and expression of faults and generates reliable probability volumes^3,11^.

Following label construction, seismic denoising is performed using Schlumberger’s machine learning–based noise attenuation module in order to enhance signal clarity before fault prediction. The self-supervised convolutional framework attenuates random noise, multiples, and acquisition artifacts that could mask reflector discontinuities. The intensity of denoising is controlled through parameters such as patch size, confidence level, and number of iterations. If filtering is too aggressive, subtle low-throw faults may be smeared or removed; if it is too weak, residual noise remains and can obscure fault features. For this reason, denoised results are validated against key horizons and known structural elements to ensure that genuine geological features are preserved while noise is effectively suppressed^8,12^.

**Fig. 4:**
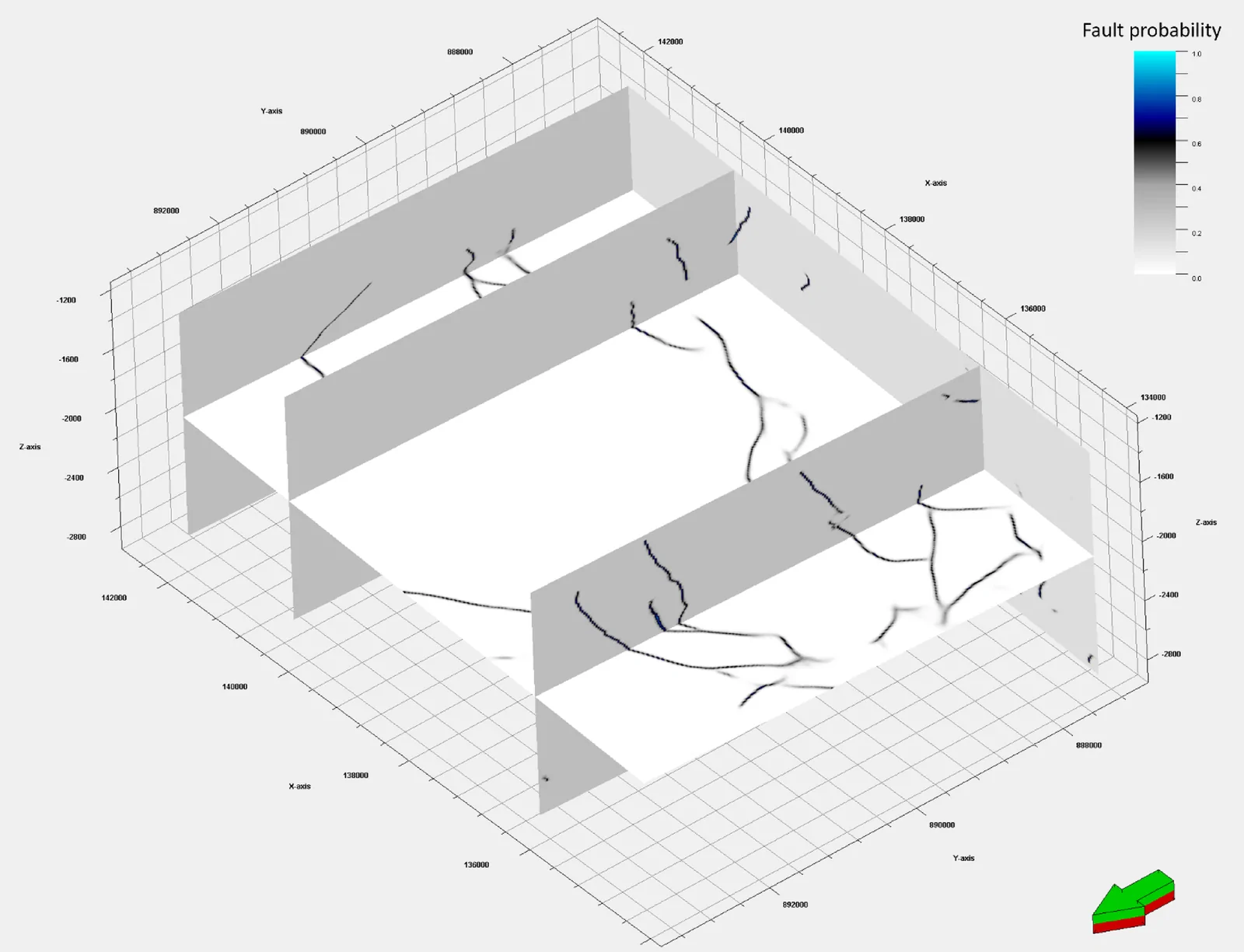
Fault-prediction cube showing faults across the area using a confidence threshold.

With denoised seismic data and reliable training labels available, the user-trained CNN fault prediction model embedded in Petrel 2025^24^ is executed to generate a fault-prediction cube that can be viewed in 3D and examined along seismic sections by ML system. The algorithm receives the full seismic cube together with the manually labeled faults and trains a U-Net style encoder–decoder network to classify each voxel as belonging either to a fault or to the surrounding background. The output of this process is a three-dimensional fault prediction attribute cube in which each voxel is assigned a confidence threshold value between zero and one. A value close to one corresponds to high model confidence that the voxel belongs to a fault, whereas values near zero indicate background (Fig. 4). This probability cube provides the quantitative foundation for the subsequent geometric analysis^1,4^.

**Fig. 5:**
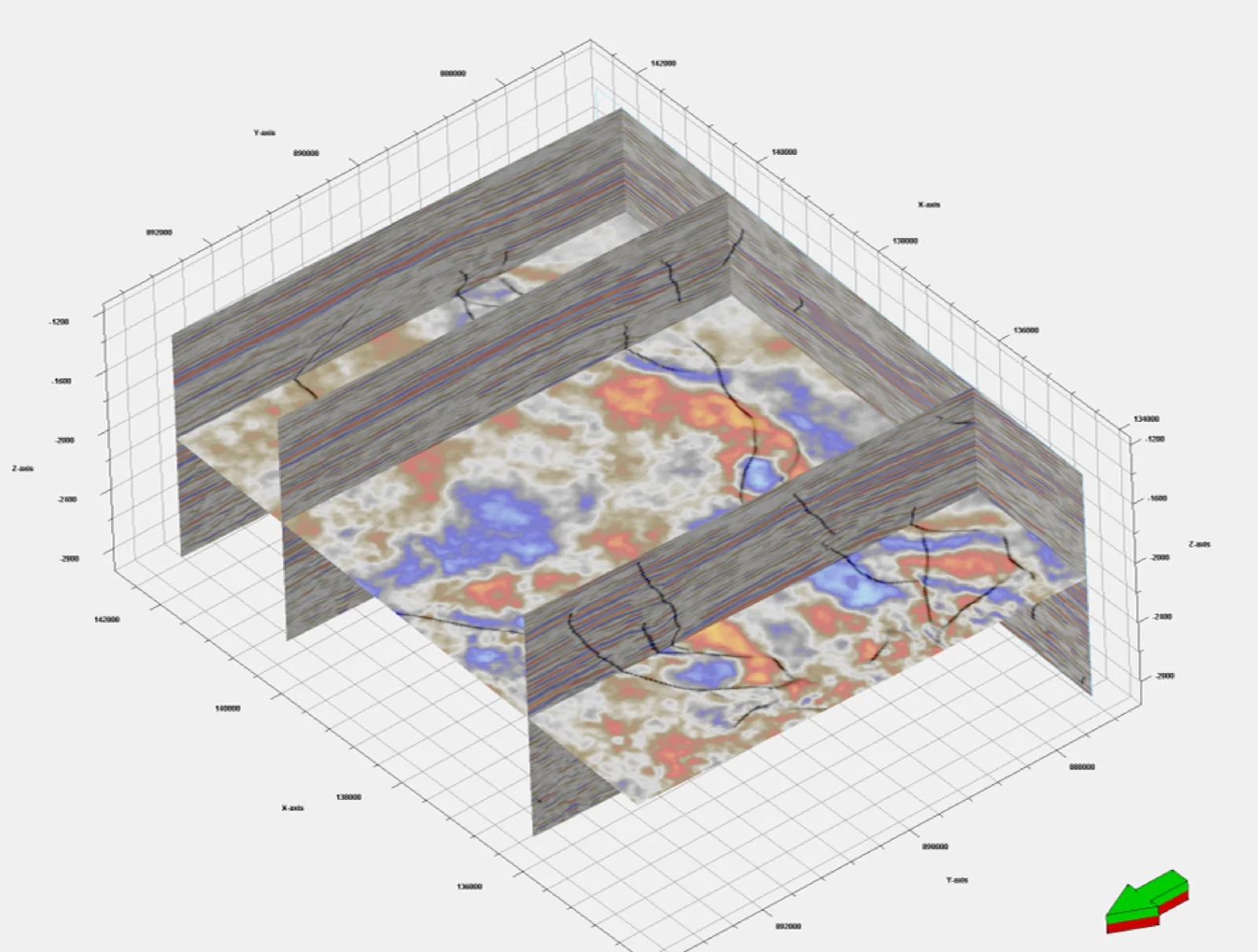
Mixer cube showing the fault-prediction cube overlaid on the seismic volume, highlighting fault probabilities.

**Prediction quality control** is then conducted to ensure geological coherence. A mixer-cube visualization overlays the machine learning–derived probability values directly onto the original seismic volume, allowing the interpreter to compare predicted fault locations with reflector terminations and discontinuities^25,26^. Any misalignments, spurious features, or missing segments identified during mixer-cube visualization guide the iterative refinement of interpreter-provided labels and extraction attributes. This refinement focuses on resolving ‘contradictory instructions’ where a fault may have been mapped on one training line but omitted on its clear continuation, causing model confusion. By adjusting these labels and re-running the prediction, the system is guided toward a more geologically coherent result. (Fig. 5)^2,3^.

**Fig. 6:**
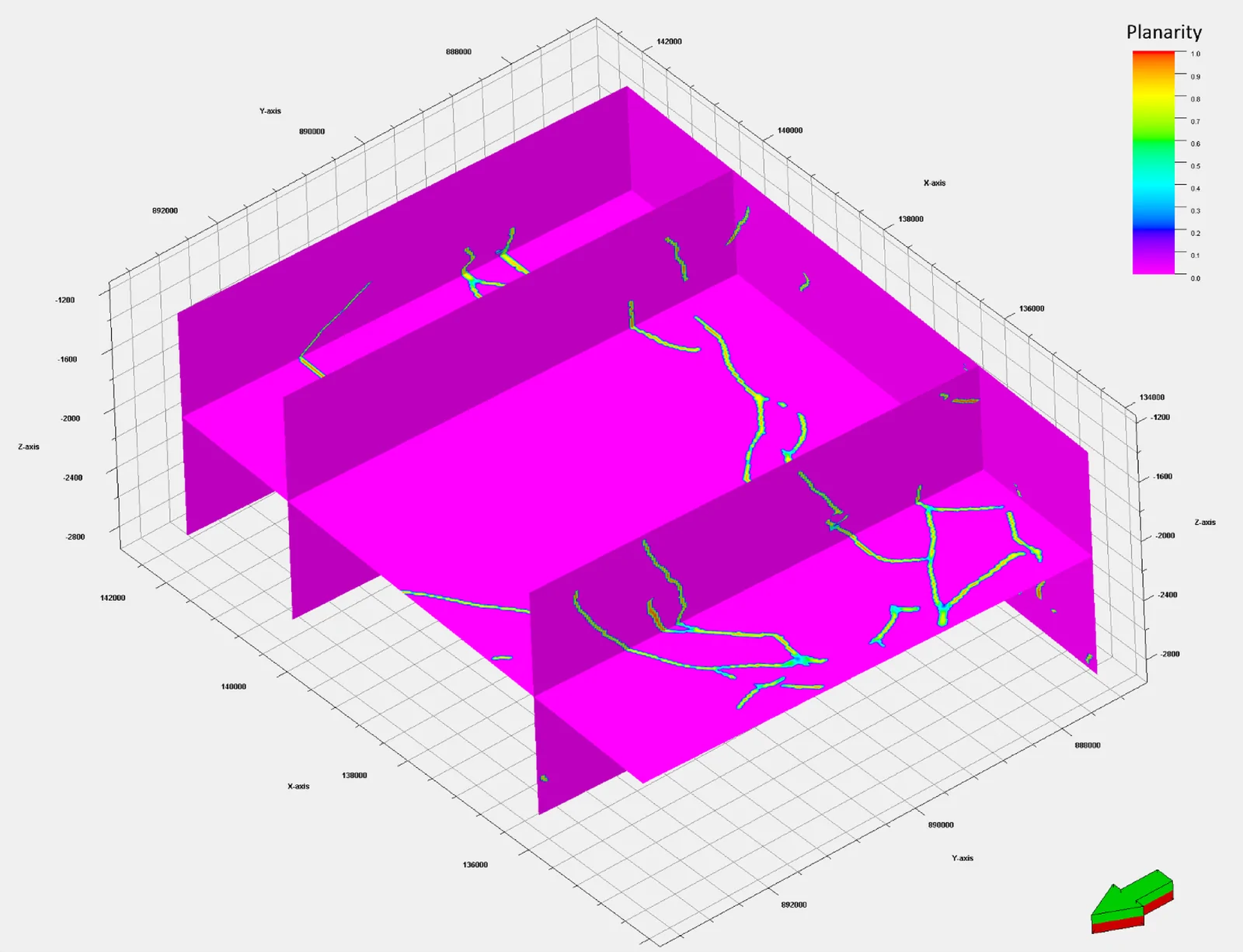
Planarity cube highlighting planar surfaces (high values) versus non-planar region.

**Fig. 7:**
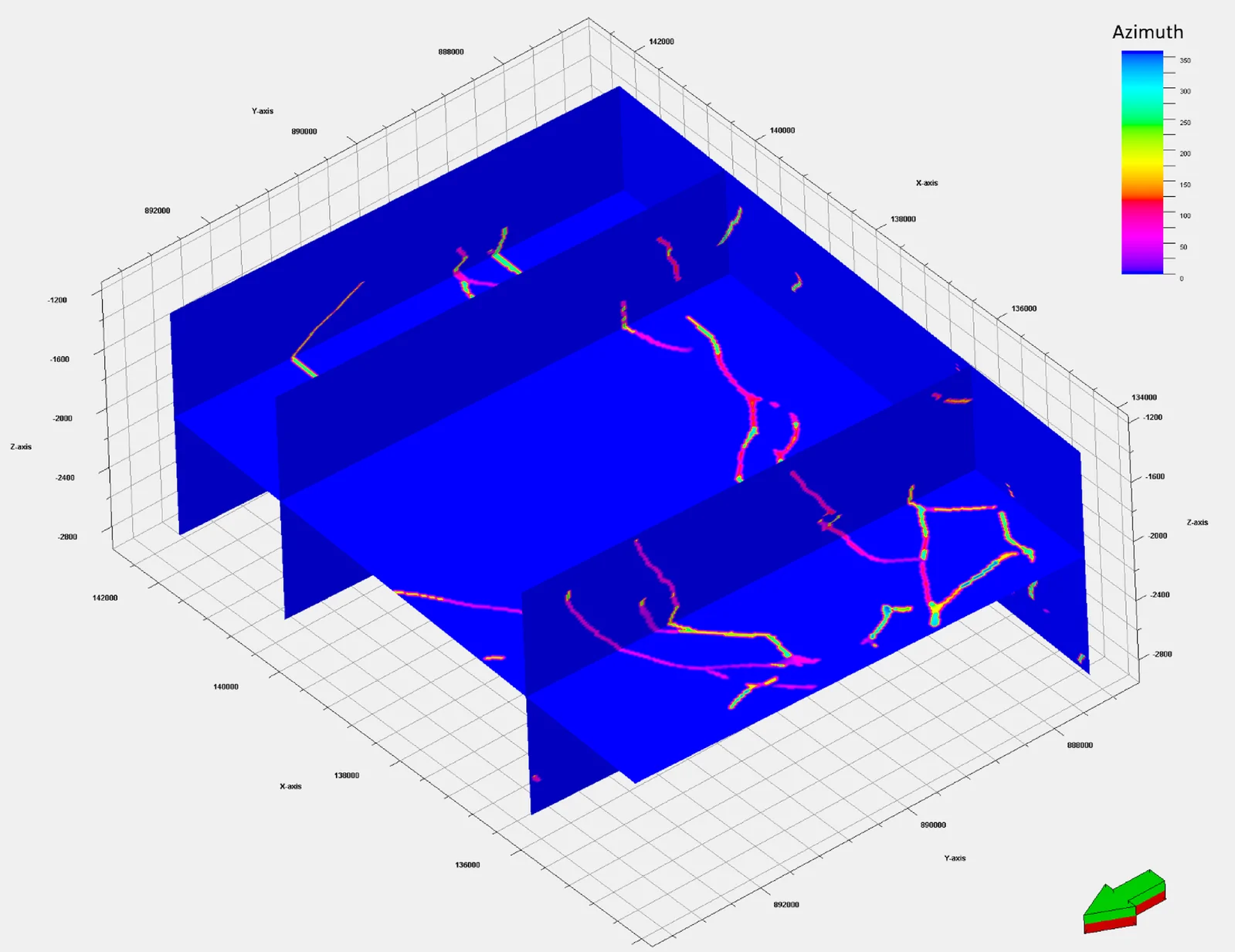
Azimuth cube highlighting the dominant orientation of features in the seismic volume.

Geometric evaluation is subsequently applied to the probability cube using the **Evaluate function**. This step generates two key attributes, planarity and azimuth (Figs. 6 and 7), that form the basis for fault segmentation^27,28^. Planarity measures how flat or sheet-like the local group of voxels is, with values approaching one indicating strongly planar surfaces such as coherent fault planes, and values close to zero indicating chaotic or non-planar regions typical of noise or stratigraphic textures. Azimuth expresses the horizontal strike direction of the fault, measured clockwise from geographic north between zero and 360 degrees, following the geological right-hand rule. Regions with stable azimuth values indicate consistent fault orientation, whereas abrupt azimuth fluctuations may signal steeply dipping surfaces or noise contamination^1,3,4^.

During the geometric evaluation stage, two primary control parameters are tuned: the minimum threshold and the radius. The minimum threshold defines the lowest probability at which a voxel is considered part of a fault. In this study, a threshold of 0.3 was found to be optimal for capturing subtle low-throw faults (< 15 ms) while suppressing artifacts. The radius parameter, which defines the 3D neighborhood used to compute planarity and azimuth (Figs. 6 and 7), was set to four voxels to provide stable attribute estimates without over-smoothing local structural details^2,5,6^.

**Fig. 8:**
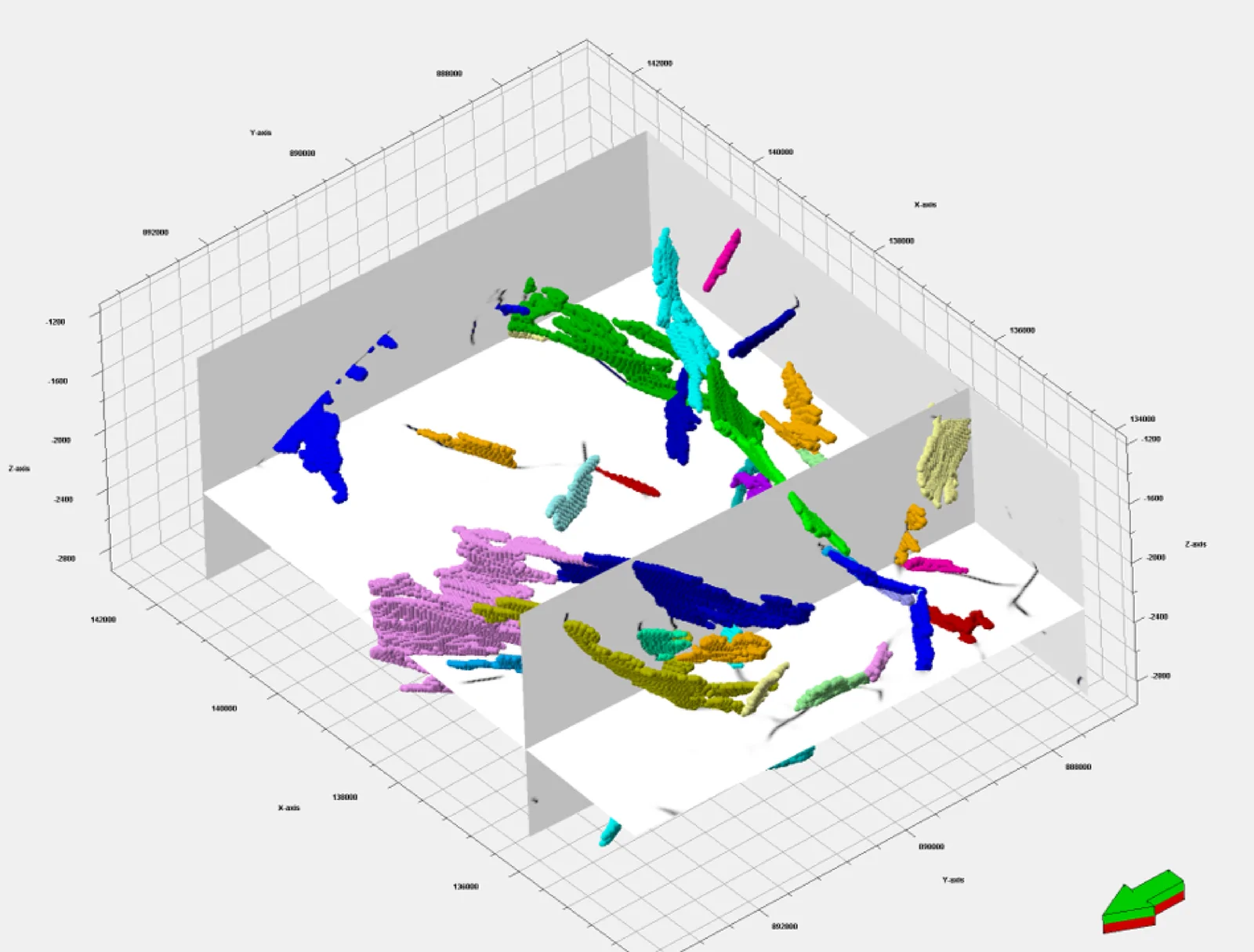
Extracted fault point sets obtained from the evaluated cubes, showing coherent fault objects filtered by probability, planarity, and azimuth thresholds.

After parameter tuning, fault extraction is performed directly from the evaluated cubes. Only voxels that simultaneously satisfy the probability threshold, exceed the selected planarity value, and fall within the desired azimuth range are retained as fault candidates. In this study, coherent fault objects were obtained using a minimum probability of 0.3, a radius of four voxels, a planarity threshold of 0.55, and an azimuth range from 0° to 359°, thereby allowing faults of all orientations to be extracted. The extraction process produces dense three-dimensional point clouds (Fig. 8) that delineate discrete fault objects while preserving the spatial resolution and geometry of the original machine learning prediction^10,11^.

**Fig. 9:**
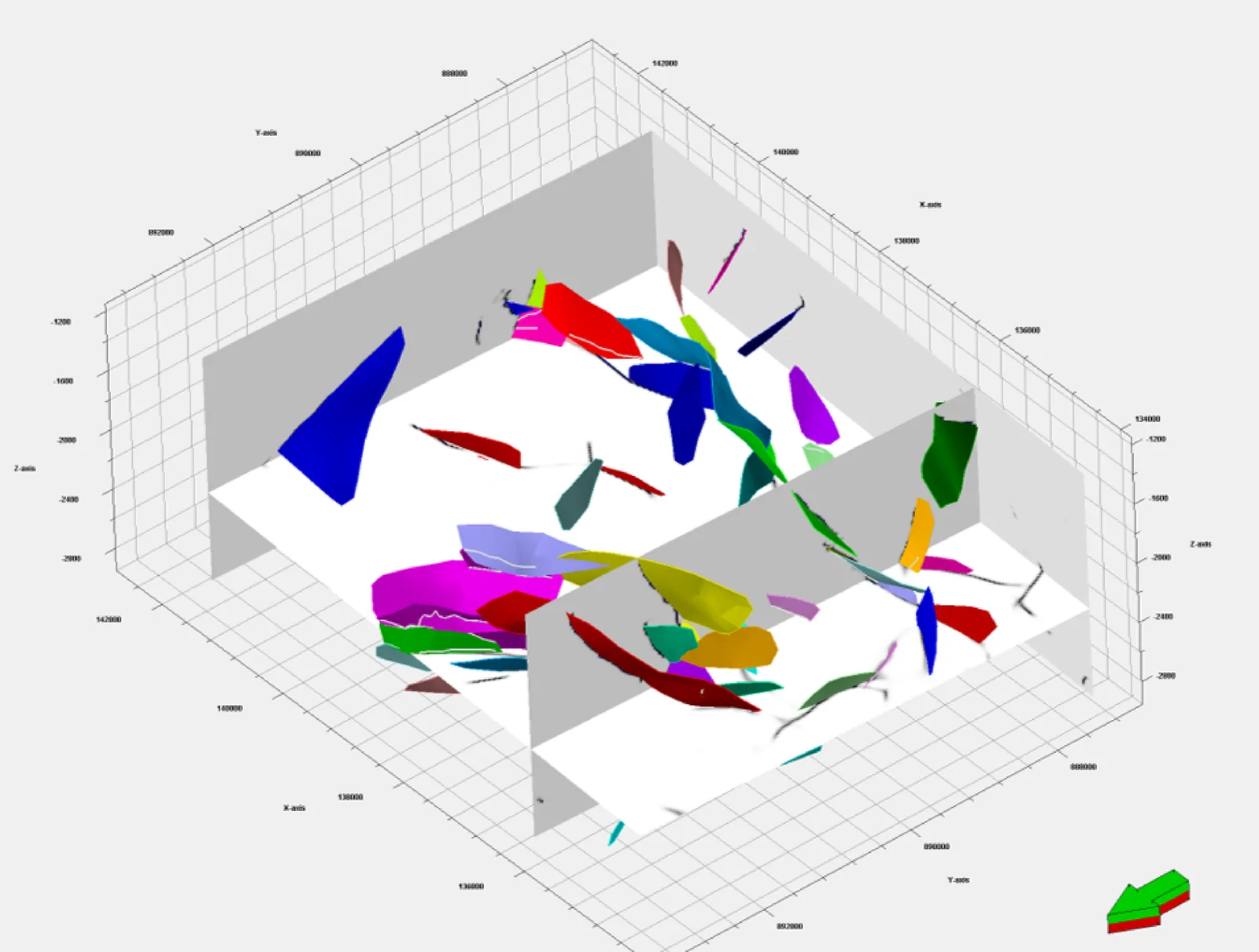
Fault planes created from triangulated point clouds, with extractable fault sticks for integration into structural framework models.

The resulting point clouds undergo systematic post-processing. Intersecting faults are split into separate objects, neighboring segments are merged where continuity is clear, artifacts and noise clusters are removed, and truncated fault traces are extended where supported by seismic evidence. Additional filtering based on azimuth consistency allows isolation of fault populations aligned with the dominant structural trends of the study area. The refined point clouds are finally triangulated or surface-fitted to generate continuous fault planes by python plug-in was developed to directly extract fault sticks from fault framework models. The resulting fault sticks are produced at uniform intervals and can be modified using standard seismic interpretation tools, thereby achieving a closed-loop integration with conventional workflows and suitable for incorporation into the structural framework model (Fig. 9)^11,12^.

To objectively assess the accuracy of the user-trained CNN model, we employed two primary quantitative metrics. First, the Dice Similarity Coefficient (DSC) was used to measure the spatial overlap between the ML-predicted fault probability volume and the manually interpreted ground-truth labels. The DSC is defined as:

$$DSC=(2\vert A \cap B\vert)/(\vert A\vert+\vert B\vert)$$

where A represents the set of voxels predicted as faults by the ML model and B represents the manually interpreted fault voxels. A DSC exceeding 0.85 was set as the benchmark for high-fidelity performance. Second, we calculated the Delineation Rate, defined as the percentage of manually mapped major faults that were successfully recovered by the automated workflow. These quantitative metrics were supplemented by qualitative visual QC using mixer cubes to ensure that the predicted discontinuities aligned with geologically sound reflector terminations.

Interpretation efficiency was evaluated by comparing the total man-hours required for the ML-assisted workflow (manual labeling of < 1% of the volume, 3D inference, and automated stick extraction) against the estimated baseline of 120 man-hours typically required for manual full-volume interpretation of similar structural complexity in the Western Desert.

## Results and discussion

The user-trained fault prediction model demonstrated exceptional performance on field-scale seismic data from the Berenice Field, effectively detecting faults with diverse structural signatures. This section presents a detailed analysis of the model’s inference results on the validation dataset and its application to the full seismic volume.

**Table 1:** Quantitative performance and validation metrics for the CNN fault model.

Metric	Result	Validation Basis
Dice similarity coefficient (DSC)	> 0.85	Voxel-wise overlap between ML predictions and manual labels on 3 training lines
Fault delineation rate	65%	Percentage of major manually mapped faults successfully recovered across the 3D volume
Time efficiency improvement	> 80%	Reduction in project turnaround time compared to traditional manual interpretation
Input training data	< 1%	Based on only 3 manually interpreted seismic lines used to train the CNN

As summarized in Table 1, the model demonstrated high fidelity in reproducing the structural framework of the Berenice Field, with quantitative benchmarks substantiating the qualitative visual agreement observed in the seismic sections.

The user-trained fault prediction model was validated by comparing ML outputs with the interpreter-guided baseline. Quantitatively, the model achieved a Dice Similarity Coefficient (DSC) exceeding 0.85 (Table 1), indicating high spatial fidelity between the CNN’s probability cube and the manual training labels. Across the full 9 × 8 km volume, the workflow successfully delineated 65% of the faults that were previously mapped manually, demonstrating its reliability in reproducing established structural frameworks. Furthermore, the model identified subtle discontinuities with throws less than 15 ms that were omitted in initial manual interpretations. These evidenced metrics confirm that the ML-assisted workflow offers an 80% reduction in interpretation time while maintaining a precision that exceeds traditional coherence-based attributes.

**Fig. 10:**
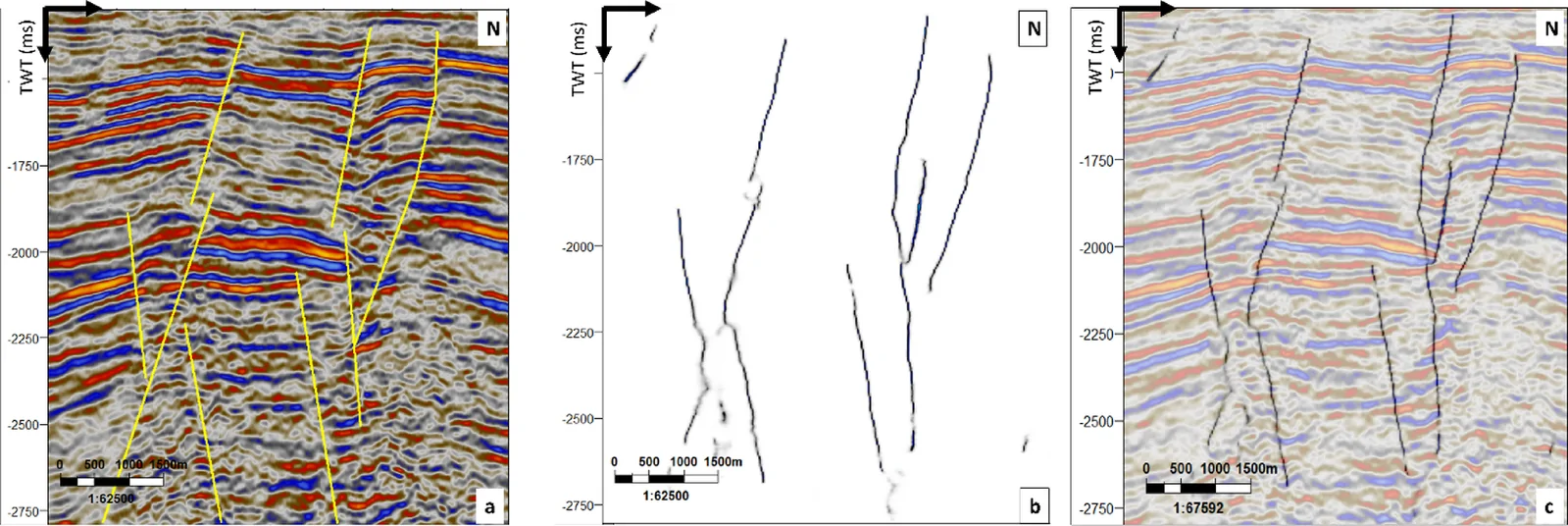
(**a**) Shows a crossline with manually picked training labels, (**b**) presents machine-learning–predicted faults along the same line, (**c**) displays Qc mixer section of seismic with faults.

Model validation was further corroborated by visual inspection of the ML predictions (Fig. 10b) against the manual interpretation subset (Fig. 10a) and the mixer cube (Fig. 10c). Across the full seismic volume, the ML system successfully captured the majority of interpretable manual faults and identified additional fault features not previously mapped. While a limited number of faults were missed in regions of poor seismic quality, these omissions are minor and do not compromise the overall structural interpretation.

Across the full seismic volume, the ML system successfully captured the majority of interpretable manual faults and identified additional fault features not previously mapped. A limited number of faults were missed in regions of poor seismic quality; these omissions are minor and do not compromise the overall structural interpretation. In some cases, major faults appeared segmented due to local noise or amplitude scattering, as shown in (Fig. 10) in the deepest southern part of the section, which can be readily addressed through post-processing to produce continuous fault planes.

The influence of training label quantity was assessed by comparing predictions using three training lines versus a larger number of labels. While increasing the number of labels generally improves lateral and vertical fault continuity, three lines were sufficient for this dataset, given the seismic resolution and structural complexity. Optimal training label requirements remain dependent on data characteristics, including signal-to-noise ratio and fault density.

ML predictions exhibit excellent continuity and enhanced geological fidelity, resolving subtle segments and splays that are often overlooked or simplified in manual interpretations. Minor discrepancies, such as localized discontinuities in intervals dominated by strong reflectors or scattered amplitudes, reflect the system’s data-driven confidence assessment and do not impede downstream extraction or structural modeling. In the upper southern part of the section and the upper northern area between the two major faults, the ML system also detected two previously unmapped minor faults, demonstrating its ability to identify subtle structures beyond the training labels (Fig. 10b, c).

**Fig. 11:**
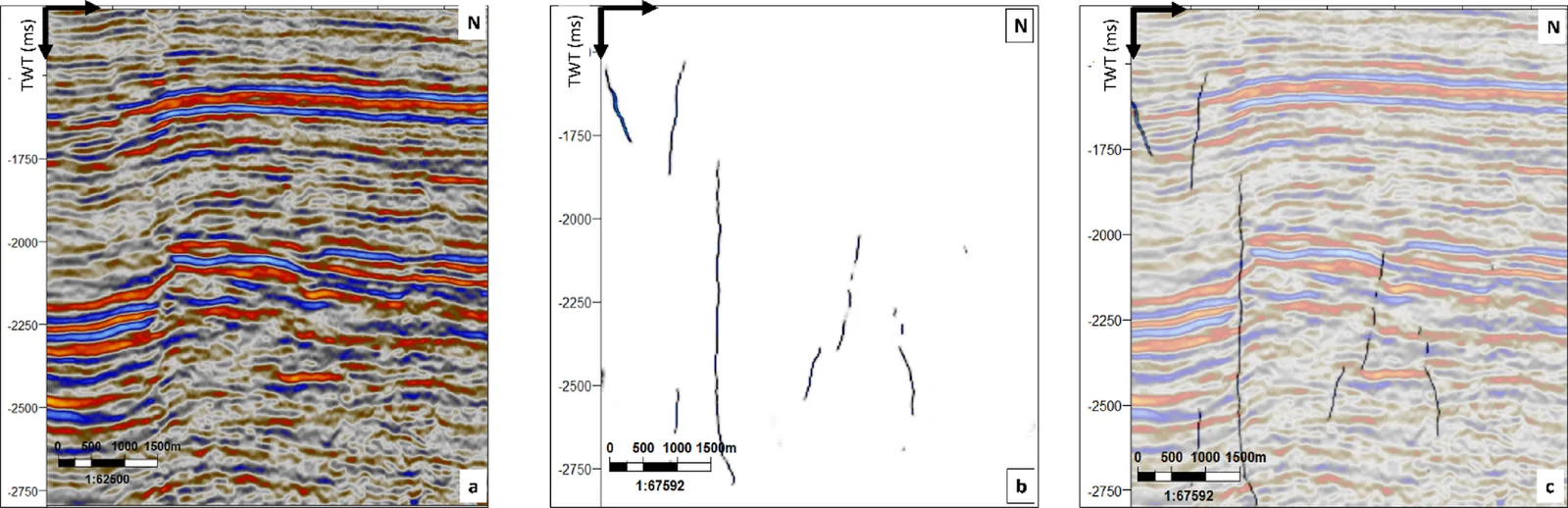
(**a**) Shows a far crossline from the training labels, (**b**) presents machine-learning–predicted faults along the line, (**c**) displays Qc mixer section of seismic with faults.

The ML system demonstrated strong capability in predicting faults in regions distant from training labels. Predictions successfully captured both major and minor fault structures, with only minor gaps caused by strong, continuous reflectors. These gaps did not compromise subsequent modeling, confirming the system’s ability to generalize beyond directly labeled areas. A seismic line located far from the training labels is shown (Fig. 11a), with the corresponding ML-predicted faults displayed (Fig. 11b). For quality control, the predicted faults were overlaid on seismic cube using mixer (Fig. 11c), showing strong agreement with the seismic data and capturing all interpretable faults. Minor segmentation observed in some faults, caused by amplitude scattering, can be effectively addressed during the extraction stage.

**Fig. 12:**
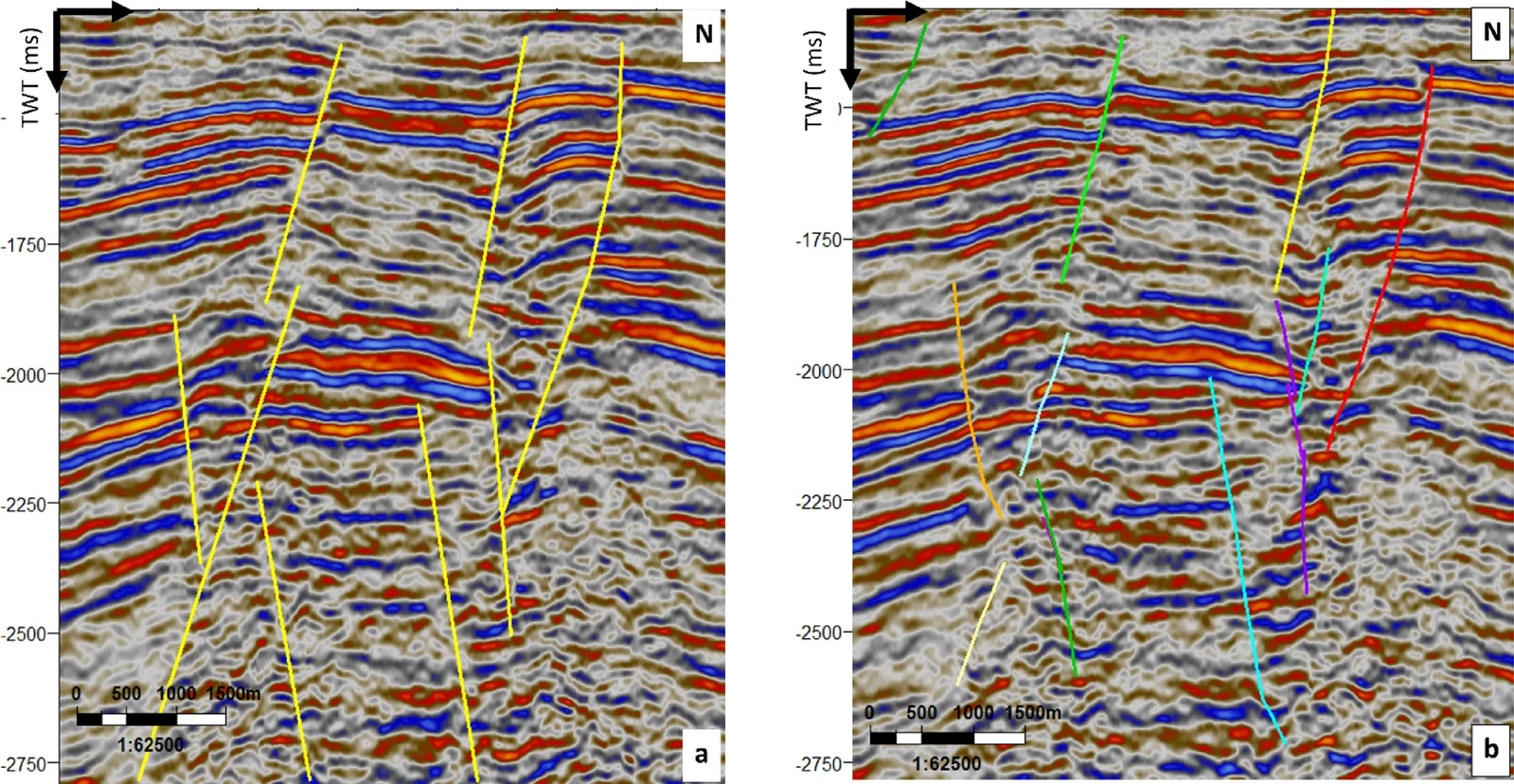
(**a**) Shows manually interpreted section, (**b**) presents machine-learning–extracted final faults along the section.

The extraction process effectively translated the high-probability voxels into coherent structural objects. A key observation was the model’s ability to maintain the primary ENE–WSW and NW–SE structural grain even in areas where amplitude scattering caused temporary segmentation of major faults. By merging these segments based on azimuth consistency, the final framework achieved a level of continuity that exceeded manual interpretations, particularly in resolving the connectivity of fault splays. Performance analysis indicates that the ML-assisted workflow successfully resolved subtle features in the upper southern and northern sectors that were initially omitted. However, a limitation was identified in the deepest southern sections, where poor signal-to-noise ratios led to localized gaps in fault planes. While these areas required minor manual edits to ensure structural closure, the overall fidelity of the automated framework remains high, as evidenced by the crossline comparison in Fig. 12. The transition from dense point clouds to extractable sticks proved to be the most significant factor in achieving the documented 80% reduction in interpretation turnaround time.

The detection of previously unmapped minor faults in the upper southern and northern sectors carries significant implications for the AEB-3E reservoir unit of the Alam El Bueib Formation. These subtle features, often with throws under 15 ms, further segment the horst and graben blocks that characterize the field’s primary structural grain. From a reservoir engineering perspective, such compartmentalization can lead to inefficient injection-production patterns if wells are placed across unrecognized barriers. By resolving these discontinuities, the ML framework enables more accurate predictions of remaining oil distribution and helps identify bypassed pay zones that traditional, more simplified manual models might overlook.

After fine-tuning the pointsets to align with the fault planes, filtering tools based on azimuth were applied to select the dominant trends in the field. The processed pointsets were then converted into fault sticks or a full fault framework using a Python plugin, enabling seamless integration into conventional interpretation workflows. The final results required only minor manual edits and demonstrated strong agreement with the manually interpreted faults, while providing enhanced detail, accuracy, and continuity. Figure 12 illustrates a crossline comparison between manual (Fig. 12a) and ML interpretations (Fig. 12b), showing no significant differences and confirming the high fidelity of the ML-assisted workflow.

The ML-assisted workflow achieved a measurable efficiency gain, with the complete structural framework produced in under 10% of the time required for a traditional manual baseline. Specifically, the manual labeling of three training lines took approximately four hours, followed by two hours of automated 3D inference and extraction. Compared to the several weeks required for exhaustive manual digitization of the 9 × 8 km volume, this represents a time reduction exceeding 80%. These observations demonstrate that the workflow successfully shifts the interpreter’s role from repetitive manual picking to high-level quality control.

## Conclusion

This study substantiates the robustness and practicality of applying Schlumberger’s CNN-driven Fault Prediction workflow for automated fault interpretation in Egypt’s Western Desert. Through its integration within the Petrel E&P environment, the methodology successfully delineated complex, multi-orientated fault systems within the Berenice Field using only minimal training data and without requiring prior machine learning expertise. The generated fault probability volumes, together with planarity and azimuth-based evaluation, provided detailed insight into subsurface fault geometries, including subtle low-throw and sub-seismic-scale features that are commonly overlooked by traditional manual workflows.

Quantitative assessments demonstrated strong agreement between ML-derived results and ground-truth manual interpretations, with over 65% of manual faults accurately reproduced and additional valid structures identified by the system. The approach drastically reduced interpretation time, improving efficiency by more than 80% while ensuring consistency and reducing interpreter subjectivity.

Overall, the integration of machine learning into seismic interpretation workflows represents a paradigm shift from manual delineation to data-driven automation. The demonstrated success in the Berenice Field validates this technique as a reliable and scalable solution for structural modeling, reservoir compartmentalization, and risk mitigation in geologically complex basins. Future applications across the broader Western Desert and analogous tectonic settings are expected to further refine quantitative uncertainty assessments and enhance the fidelity of regional geological models.

## Data Availability

Data are however available from authors upon reasonable and with permission of [The Egyptian General Petroleum Cooperation].
